# Antiviral Therapy by HIV-1 Broadly Neutralizing and Inhibitory Antibodies

**DOI:** 10.3390/ijms17111901

**Published:** 2016-11-18

**Authors:** Zhiqing Zhang, Shaowei Li, Ying Gu, Ningshao Xia

**Affiliations:** 1 State Key Laboratory of Molecular Vaccinology and Molecular Diagnostics, School of Public Health, Xiamen University, Xiamen 361102, China; zhiqingzhang.xmu@foxmail.com (Z.Z.); shaowei@xmu.edu.cn (S.L.); nsxia@xmu.edu.cn (N.X.); 2 National Institute of Diagnostics and Vaccine Development in Infectious Disease, School of Life Sciences, Xiamen University, Xiamen 361102, China

**Keywords:** HIV-1, broadly neutralizing antibody, epitope, antibody therapy

## Abstract

Human immunodeficiency virus type 1 (HIV-1) infection causes acquired immune deficiency syndrome (AIDS), a global epidemic for more than three decades. HIV-1 replication is primarily controlled through antiretroviral therapy (ART) but this treatment does not cure HIV-1 infection. Furthermore, there is increasing viral resistance to ART, and side effects associated with long-term therapy. Consequently, there is a need of alternative candidates for HIV-1 prevention and therapy. Recent advances have discovered multiple broadly neutralizing antibodies against HIV-1. In this review, we describe the key epitopes on the HIV-1 Env protein and the reciprocal broadly neutralizing antibodies, and discuss the ongoing clinical trials of broadly neutralizing and inhibitory antibody therapy as well as antibody combinations, bispecific antibodies, and methods that improve therapeutic efficacy by combining broadly neutralizing antibodies (bNAbs) with latency reversing agents. Compared with ART, HIV-1 therapeutics that incorporate these broadly neutralizing and inhibitory antibodies offer the advantage of decreasing virus load and clearing infected cells, which is a promising prospect in HIV-1 prevention and treatment.

## 1. Introduction

Since 1983, human immunodeficiency virus type 1 (HIV-1) has been a worldwide epidemic, with reportedly more than 35 million people living with HIV-1 globally and 2.1 million newly infected each year. HIV-1 infection causes acquired immune deficiency syndrome (AIDS), which is characterized by a significant reduction in CD4^+^ T cells and damage to the immune system. The current treatment strategy for HIV-1 infection is antiretroviral therapy (ART). While most antivirals against HIV-1 approved by the Food and Drug Administration are reverse transcriptase inhibitors (RTIs) and protease inhibitors (PIs), the integrase inhibitors (INIs) have become a prominent class of antivirals [1]. Because long-term treatment frequently leads to resistance and side effects in patients, inhibitors that target other critical steps in the virus replication cycle are vital and are in various stages of development. Of interest are fusion inhibitors, which target the fusion process. For example, T20 is the first FDA-approved fusion inhibitor that can bind to gp41 protein to block viral infection [2,3].

Although ART can suppress the HIV-1 replication cycle and control the disease process, it cannot completely clear any existing viral reservoirs. As such, an ideal way to prevent HIV-1 infection would be through the use of a vaccine. However, this approach is also plagued by specific challenges and, after more than three decades, HIV-1 vaccine development is still “on the way”. B-cell targeting vaccines have proved disappointing, with two phase III gp120 vaccine trials (VAX003 in Thailand and VAX004 in the North America) showing no vaccine efficacy [4,5]. This failure saw a shift in the focus away from B-cell targeting to T-cell targeting strategies. However, two Phase IIb trials (STEP and Phambili) not only failed to show any efficacy but also demonstrated the increased risk of HIV-1 acquisition among vaccine recipients as compared with the placebo group [6,7,8]. With these failures, the prospects for the success of a HIV vaccine seemed poor.

The Thai phase III trial (RV144) was a randomized, multicenter, double-blind and placebo controlled efficacy trial undertaken in Thailand. The RV144 vaccine showed 31.2% protection from HIV-1 infection over a period of 42 months, the first vaccine ever to show such results [9]. This result was unexpected, as the trial was initially criticized because the vaccination regimen included the AIDSVAX B/E gp120 vaccine, which had previously been shown to be ineffective. The study combined the AIDSVAX with the CD4+ T-cell stimulating ALVAX canarypox vaccine, and this combination appeared to offer a protective effect (albeit, a mild one), with correlations between the plasma concentration of IgG antibody specific for the V1V2 loop and protection, and between the IgA antibody and the risk of infection [10]. Quite how the RV144 vaccine exerts its effect is still unclear, but these findings have been used as a basis for new clinical trials that optimize different strategies; for example, incorporating the clade C recombinant gp120 and different adjuvants. Despite these successes, however, an effective HIV-1 vaccine is still sometime away from fruition.

Since most licensed vaccines can be functionalized with neutralizing antibodies (NAbs), inducing NAbs in individuals could be key in the development of a successful HIV-1 vaccine [11]. Indeed, studies have shown that 20% to 30% of HIV-1-infected patients develop broadly neutralizing antibodies (bNAbs) that target various strains of the virus during chronic infection [12,13]. The immunogen to induce the production of NAbs is the HIV-1 envelope glycoprotein spike (Env), a heavily glycosylated trimer containing heterodimers of gp120 and gp41. The Env protein is a major structural protein involved host cell penetration that protrudes from the viral membrane. For HIV, a successful bNAb would be able to recognize this known diversity of Env. To date, however, there have been no Env-based vaccines to induce bNAbs, and this has been attributed to the conformational flexibility, sequence variability and glycan shielding in the Env trimer [14].

In recent years, with the development of single B-cell sorting and deep sequencing, abundant bNAbs have been isolated from HIV-1-infected donors, and their potency and epitopes have been extensively studied [15]. However, viruses and NAbs co-evolve, and the mature bNAbs have unusual characteristics, including highly somatic hypermutations, extremely long heavy-chain complementarity determining region 3 (HCDR3) loops, and polyreactivity [14]. Furthermore, because of the high variability of HIV-1 Env and the unique properties required of a bNAb, the development of rare bNAbs for chronically HIV-1-infected individuals may take several years. Such bNAbs will not only play a significant role in guiding the new structure-based immunogen designs but also contribute to both HIV-1 prevention and treatment fields.

Pre-exposure prophylaxis (PrEP) is an important tool for the prevention of HIV/AIDS in people who do not have HIV/AIDS but who are at a substantially higher risk of contracting an HIV infection, and it can be mediated by ART and bNAbs. Compared with ART, PrEP mediated by bNAb can neutralize the free virus before the infection and act earlier than antivirals. With respect to antibody therapy, numerous pre-clinical and clinical trials are performed to evaluate their potential therapeutic efficacy. In latest progress on anti-HIV therapy, Byrareddy et al. reported that the combination of antiretroviral and α_4_β_7_ antibody therapy could effectively control viremia and reconstitute immune systems in macaques infected with simian immunodeficiency virus (SIV). Intriguingly, this occurred during the combinatorial treatment period and even after the treatment was terminated [16], indicating that the antibody is targeting the integrin in CD4^+^ T cells (α_4_β_7_) instead of bNAbs directed to the virus per se. The authors identified a series of correlates that may contribute to the reduced damage observed in gastrointestinal tissues (GITs), and may control viremia in the combination therapy. Thus, bNAbs and other antibodies with HIV-1 inhibitory activity might be more advantageous than anti-HIV.

## 2. Major HIV-1 Env Epitopes and Their Corresponding bNAbs

**Figure 1:**
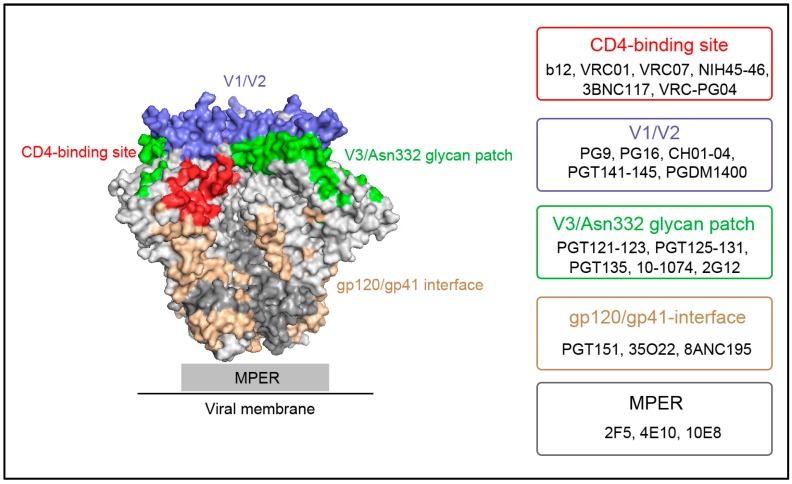
The human immunodeficiency virus type 1 (HIV-1) broadly neutralizing antibodies (bNAbs) epitopes on envelope glycoprotein spike (Env) trimer. The surface representation of Env is derived from the crystal structure of BG505 SOSIP.664 (PDB no. 4TVP). Major HIV-1 Env epitopes are highlighted in different colors (CD4bs (red), V1/V2 (tv-blue), V3/Asn332 glycan patch (green), gp120/gp41-interface (wheat), MPER (denoted as gray rectangle)) and their representative bNAbs are listed in the right boxes. Other unspecified regions are in light grey for gp120 moiety and in dark grey for the gp41 moiety. The structural figure was generated with the program PyMOL (http://www.pymol.org/).

Over the past several decades, antibodies to HIV were produced through electrofusion hybridoma or random cloning of heavy and light chains in phage display libraries, and, until recently, there were only four well-defined bNAbs: 2G12, b12, 2F5 and 4E10. In recent years, new techniques have emerged to identify bNAbs from HIV-1-infected donors. Early work by Scheid and colleagues established a flow cytometry method to identify and enrich HIV-1-specific memory B cells from the blood samples of patients infected with HIV [17]. Notably, Walker and colleagues developed a high-throughput strategy combining ELISA and neutralization to analyze the culture supernatants of about 30,000 activated memory B cell clones, and isolated several potent bNAbs [18,19]. Thereafter, based on the abovementioned method of Scheid and colleagues, Wu and colleagues designed a rational CD4 binding site mimic antigen—dubbed resurfaced stabilized core 3 (RSC3)—which could be used to identify B cells from HIV-1-infected individuals whose sera contained bNAbs [20]. The bNAbs can be classified into five subclasses, based on the recognition epitopes (Figure 1) [21]. These subclasses of bNAbs will be explored separately in the following section.

### 2.1. CD4-Binding Site

The CD4-binding site is a conserved and conformational epitope responsible for CD4 receptor binding. b12 was the first CD4-binding site bNAb to be identified, but this antibody shows limited breadth and potency (about 40% of HIV-1 strains) [22]. Later studies identified a range of other bNAbs, including VRC01, VRC07, NIH45-46, 3BNC117, and VRC-PG04, among others, which also target the CD4-binding site but have potent and broadly neutralizing activity [20,23,24,25]. For example, VRC01 can neutralize 91% of 190 pseudoviruses with a 0.33 μg/mL geometric mean IC_50_ (Table 1) [20]. Given their potency, the VRC01-class of bNAbs were further explored using next-generation sequencing, from which the findings revealed a set of “VRC01 signatures” characterized by a high degree of somatic hypermutations, a distinctive five amino acid light-chain complementarity determining region (CDRL3), and a preferential germline allele of the heavy chain (IgHV1-2*02) [26]. Given that multiple donors can develop VRC01-class bNAbs, it has been suggested that the human immune system can recognize vulnerable CD4-binding site. Thus, the CD4-binding site on the Env trimer could be an ideal target for vaccine design.

### 2.2. First and Second Variable Domains (V1/V2)/Asn160 Glycan Dependence

PG9, PG16, CH01-04, PGT141-145, and PGDM1400 are among a class of bNAbs that recognize the first and second variable (V1/V2) domains of the Env trimer [18,19,27,37]. This epitope involves the trimer apex of the Env quaternary structure. Because the V1/V2 domains are highly variable—presumably to evade neutralization—the bNAbs have evolved a special and long CDRH3 “hammer-head” structure that enables antibodies to bind to the underlying protein surface (residues 165–171, as well as an Asn160 glycan [38,39]). Another antibody, PGDM1400, was isolated from a chronically infected donor using the recombinant HIV-1 envelope trimer, BG505 SOSIP.664 gp140 as an affinity antigen. PGDM1400 is since regarded as one of most potent bNAbs discovered to date, bearing a broad (83%) and potent cross-clade neutralizing activity with a median IC_50_ of 0.003 μg/mL (Table 1) [27].

### 2.3. Third Variable Domain (V3)/Oligomannose Glycan Patch Involving Asn332 Glycan

The V3-glycan epitopes are regarded as the “supersite of vulnerability” and depend on the N332 glycan [40], with several bNAbs identified to date (PGT121–123, PGT125–131, PGT135, 10-1074 and 2G12) [28,40,41,42]. 2G12 was the first glycan-dependent bNAb to be identified, and it has a unique domain-swapped antibody structure to facilitate oligomannose glycan recognition [43,44]. Structural resolution analyses have shown that, of the list of candidates, PGT121 is not inclusively dependent on N332 glycan recognition [28,29]. Intriguingly, these bNAbs bind to an epitope that involves the V3 conserved base, which comprises a GDIR peptide motif [19]. On the other hand, PGT135 targets a more complex epitope that consists of multiple glycans and the underlying part of the V4 loop [40]. A glycan cluster is a target of humoral immune recognition, and a glycan cluster-shielded Env trimer is one of the classical viral evasion mechanisms [45]. Targeting these epitopes is another potential avenue for vaccine development.

### 2.4. gp120/gp41 Interface

PGT151, 35O22, and 8ANC195 are recently isolated bNAbs that target the gp120–gp41 interface epitope, which is specific for native-like Env trimers. PGT151 was isolated by direct functional screening from an “elite controller”, who was infected with the clade C virus. Elite controllers have a low-to-undetectable viral load using RNA assays, and account for 0.5% to 1% of individuals with HIV-1 [46]. PGT151 recognizes a glycan-dependent epitope on cleaved envelope trimers and stabilizes Env on a pre-fusion conformation [30,31]. By comparison, 35O22 and 8ANC195 recognize partially overlapping epitopes that are different from that of PGT151:35O22 was shown to neutralize 62% of 181 pseudoviruses with 0.056 μg/mL geometric mean IC_50_ (Table 1) [32], whereas 8ANC195 anchors the partially open state of the Env glycoprotein, and can neutralize 57% of diverse viruses [33,34]. In contrast, a recent study by Kong and others found that the N123-VRC34.01 neutralizing antibody acts by binding to the N-terminal 8 residues of the gp41 fusion peptide and glycan N88 of gp120 [47].

### 2.5. Membrane Proximal External Region (MPER)

MPER is a highly conserved region of gp41 and plays an important role in the viral fusion process. The bNAbs 2F5 and 4E10 recognize continuously linear epitopes (ELDKWA and NWFNIT, respectively) within this region [48,49,50]. However, both bNAbs are autoreactive, which enhances the binding of antibodies to the MPER of gp41 by reacting with lipid membrane. More recently, a new MPER antibody, 10E8, was isolated, and this antibody shows better efficacy than 2F5 and 4E10 in terms of its breadth and potency in vitro neutralization activity [35]. Unlike to 2F5 and 4E10, 10E8 is not autoreactive but targets a structural site comprising hydrophobic residues within the 671–683 stretch of MPER and a critical arginine or lysine residue located before the transmembrane region [35,36]. The highly conserved MPER is a vulnerable site, and thus a potentially valuable tool for HIV-1 vaccine design for the induction of neutralizing antibodies.

## 3. Antiviral Therapy by Anti-CD4/HIV-1 Broadly Neutralizing Antibodies

It is challenging to overcome the potential roadblocks in effective HIV-1 vaccine design. In recent years, several bNAbs have been offered as alternative approaches for HIV-1 therapy during chronic infection. Whereas ART mainly interferes with the virus life cycle to prevent replication, bNAbs directly neutralize the free virus and target and kill the infected cells, possibly through an Fc receptor-mediated effector mechanism.

Early studies revealed that Ibalizumab (also known as TNX-355 and Hu5A8), an anti-CD4 humanized monoclonal antibody, was potently antiviral, as it blocked HIV-1 entry into the cell [51,52]. The crystal structure of Ibalizumab-Fab in complex with the first two domains (D1–D2) of CD4 showed that the binding site of Ibalizumab was located within residues 121–125 of D2, and that this did not interfere with its binding to major histocompatibility complex class II (MHC II) molecules [53]. Our group isolated a CD4 domain-1-specific monoclonal antibody (named 15A7) that also showed broad inhibition to a range of primary HIV-1 isolates and T cell-line passage strains [54]. Therefore, Ibalizumab and 15A7 exerted their antiviral effect by preventing the CD4-bound gp120 from interacting with its co-receptor [54,55]. Ibalizumab earlier clinical trials noted that it was safe, well tolerated and offered significant antiviral efficacy in HIV-1 infected adults [56]. Subsequently, an Ibalizumab-based regimen was used to treat a 56-year-old male who was infected with HIV-1 and who had high-level resistance to all five classes of antiretrovirals. The results showed that the viral load was reduced by about 4.0 log_10_; however, an inadvertently missed infusion caused rapid viral rebound and the patient developed resistance to the drug [57]. The breadth and potency of Ibalizumab against HIV-1 were 92% and 0.03 μg/mL median IC_50_, respectively. However, others have shown that the absence of the V5 glycan in Env can cause natural resistance [58], which would render the drug ineffective.

The protective efficacy of neutralizing monoclonal antibodies to HIV-1 Env are higher than those for the CD4 receptor in vivo [59]. 2G12, 2F5 and 4E10 antibodies have been assessed in clinical trials, with, for example, the potency of a triple-bNAb cocktail tested in six acutely and eight chronically HIV-1-infected individuals who underwent ART interruption. The authors found that neutralizing antibody therapy following ART cessation delayed the HIV-1 rebound as compared with a control group, not treated with antibody therapy, and further supposed that this eventual rebound was caused by the development of viral escape mutants to 2G12 (which provides most of the in vivo efficacy of the triple-bNAb cocktail) [60]. Recently, in a rhesus macaques and humanized mice mucosal challenge model, VRC01 showed potent and broadly neutralizing activity and protection from virus infection [60,61]. In a phase I clinical trial conducted by the Vaccine Research Center (VRC), National Institute of Allergy and Infectious Diseases (NIAID) at the National Institutes of Health (NIH), VRC01 showed its safety and tolerability in healthy adults [62]. The same was true when VRC01 was administered during chronic HIV-1 infection [63]. In this study, 27 subjects were allocated to five groups, with blood samples collected at different time points to assess the impact of (1) two VRC01 infusions on the persistent HIV-1 reservoir size and (2) a single VRC01 infusion on plasma viremia suppression. The results showed that infusion with VRC01 was safe, well tolerated and long lasting in subjects on and off ART. However, infusion with two doses of VRC01 was unable to reduce the cell-associated virus load in HIV-1-infected subjects receiving effective ART with undetectable plasma viremia [63]. An alternative interpretation was that the treatment with VRC01 was only short term, and that the cell-associated reservoir was in a latent state. In terms of the single VRC01 infusion arm, six of the eight ART-untreated, viremic subjects who had been infected with a circulating VRC01-sensitive virus showed a clear effect on plasma virus load. The other two subjects showed a minimal response, suggestive of a predominantly VRC01-resistant virus prior to the treatment [63]. The authors further investigated the phenotypic and genetic changes to the virus after VRC01 infusion in samples from four donors who displayed only partial suppression of plasma viremia. In these samples, there was a preferential selection and outgrowth of viruses with less sensitivity to VRC01 [63]. Overall, these findings show that, despite the positive suppression effect of VRC01 infusion, it is important to consider other factors, such as antibody concentration, virus selection, pre-infusion virus load, and viral sensitivity for the effective prevention and treatment of HIV-1 infection.

Another VRC01-like antibody, 3BNC117, also has a suppressive effect in individuals infected with HIV-1. In a phase I clinical trial of 12 uninfected and 17 HIV-1-infected individuals, 3BNC117 infusion was determined to be safe, well tolerated and effective in reducing HIV-1 viremia [64]. Furthermore, a more recent report suggests that HIV-1 immunotherapy with 3BNC117 elicits host immune responses in viremic subjects [65]. Infusion with 3BNC117 can reportedly increase the neutralizing activity to heterologous tier 2 viruses as compared to untreated individuals. Others suggest that 3BNC117 not only clears the free virus and blocks new infection but also enhances the elimination of HIV-1-infected cells [66]. In a Phase IIa clinical trial, 3BNC117 was evaluated in 13 HIV-1-infected individuals for whom treatment was interrupted [67]; the virus load will rebound rapidly when ART is disrupted in HIV-1 infected individuals. However, the trial showed that 3BNC117 infusion could delay viral rebound for longer during regular ART interruption [67], which suggests that latent reservoirs of HIV-1 remain controlled by 3BNC117. 

The 10-1074 antibody is extraordinarily potent, has a wide neutralizing activity against the V3 loop, and is strictly dependent on the glycosylation of Asn332 [28]. Shingai and colleagues reported that 10-1074 could rapidly reduce virus load in macaques chronically infected with simian-human immunodeficiency virus (SHIV); however, the subsequent emergence of neutralization-resistant variants led to virus rebound. Interestingly, the administration of 10-1074 along with 3BNC117 can suppress the plasma viremia for longer [68], with others reporting that 10-1074 has a protective effect against SHIV challenge (median = 12.5 weeks) in a macaque model [69]. A phase I clinical trial of the 10-1074 is currently ongoing to evaluate the safety, pharmacokinetics, and antiretroviral effects of the compound in HIV-1-infected and -uninfected subjects. Likewise, a phase Ib clinical trial is at the stage of participant recruitment, and it aims to evaluate the combination of 10-1074 and 3BNC117 therapy in HIV-1-infected individuals.

ART comprises a combination of several antiretroviral agents. Similarly, antibody-drug and antibody-antibody combinations will likely be needed to control HIV-1 infection effectively. It is therefore likely that novel strategies will be needed to improve the efficacy of anti-HIV therapy. Below, we highlight some of these potential avenues. 

## 4. Novel Strategies to Improve the Therapeutic Efficacy

Since 2009, numerous highly potent and broadly neutralizing antibodies targeting different epitopes of Env have been discovered, and it has been proposed that the potential combinations of different antibodies will work favorably in HIV-1 prevention and treatment. Indeed, double-, triple-, and quadruple-monoclonal antibody (Mab) combinations have been used to substantially improve the neutralization breadth of the treatment, as compared with the delivery of single Mab [70]. Furthermore, three distinct patterns have been observed among Mabs, with additive, synergistic or potency-dependent effects. Using a mathematical model to predict neutralizing activity, Wagh and colleagues found that triple and quadruple combinations were more effective than double combinations against HIV-1 [71], and this might provide clues for the development of antibody combinations for clinical trials.

Engineered, bispecific antibodies are also promising candidates for HIV-1 prevention and therapy. In 2013, Pace and colleagues created bispecific antibodies that combine Ibalizumab (iMab) with anti-gp120 bNAbs [72]. Two of these bispecific bNAbs, PG9-iMab and PG16-iMab, showed exceptional anti-HIV-1 activity through a synergistic potency caused by an increase in the local concentration of PG9/PG16 at its target cell surface as well as CD4 anchoring mediated by iMab [72]. Other bispecific antibodies that combine either anti-CD4 (iMab) or anti-CCR5 (P140) with gp41-MPER-specific bNAb (10E8) also showed potent and broad HIV-1 neutralization [73]. The authors reported that 10E8_v2.0_/iMab was the most potently and broadly neutralizing antibody. The antibody has a neutralization activity with an IC_50_ of 0.002 μg/mL in the 118 HIV-1 pseudovirus neutralization assay and a breadth of 99% in a panel of 200 HIV-1 isolates (covering additional clade C isolates than those in neutralization assay). Interestingly, the 10E8_v2.0_/iMab showed a longer reduction in virus load in HIV-1-infected humanized mice than previous bNAbs studies [73]. Bournazos and colleagues took a different approach, and combined 3BNC117 (CD4bs epitope) with PGT135 (gp120 V3-glycan epitope) through an engineered hinge domain version of IgG3 to enhance Fab domain flexibility, which was necessary for hetero-bivalent binding to the Env trimer [74,75]. The 3BNC117/PGT135 IgG3C biNAb showed synergistic neutralization potency in vitro and enhanced therapeutic activity in vivo in HIV-1 infected humanized mice [75].

Antibodies can also be modified to have longer half-lives in vivo. VRC01-LS is a mutated version of VRC01 with a higher affinity for FcRn and a three-fold longer half-life in vivo. It has also been reported to offer superior protection against SHIV infection as compared with VRC01 in rhesus macaques [69,76]. Considering these advantages, a phase I clinical trial for VRC01-LS has been launched to investigate: (1) its safety and pharmacokinetics in healthy adults in a dose-escalation study; (2) its safety and virologic efficacy in HIV-infected adults in a single-dose study; and (3) its safety, pharmacokinetics and anti-viral activity in the serum and mucosa of healthy, HIV-infected adult participants.

Despite all these promising candidates, HIV-1 infection can develop a viral reservoir in infected subjects, and persistent ART still cannot eliminate the reservoir of latently infected cells. An approach was proposed to eradicate this reservoir by combining bNAbs with latency reversing agents (LRA) [77]. Halper-Stromberg and colleagues examined the efficacy of bNAbs/LRA treatment in HIV-1-infected humanized mice administered with 3BNC117, 10-1074 and PG16 together with vorinostat (histone deacetylase inhibitors, HDACi), I-BET151 (BET protein inhibitor) and CTLA4 (a T cell inhibitory pathway blocker). The authors found that these inducers help bNAbs to effectively decrease the reservoir while monitoring the viral rebound [78]. The strategy of combining bNAbs with LRAs should be emphasized in forthcoming clinical trials.

## 5. Perspective

Nowadays, with the advent of new, potent bNAbs, HIV-1 vaccine development has been reignited with HIV-1 immunogen designs. However, the co-evolution of virus and antibody within such a complex immune system as the human body means that immunogen designs are complicated. Thus, HIV-1 vaccine research is still challenging. Recent years have seen remarkable improvements in passive therapy, and the therapy by bNAbs has revealed a novel and effective strategy for HIV-1 suppression. Clinical trials of Ibalizumab, VRC01 and 3BNC117 have highlighted that bNAbs are safe and well tolerated in patients, but that they also suppress the virus load. In addition, bNAb combinations and bibNAbs are promising candidates that will enhance the potency and breadth of antibody therapies. It is undoubted that relevant bNAbs have already entered the clinical trials in the search for effective candidates for HIV-1 therapy.

## Figures and Tables

**Table 1:** Characteristics of the representative broadly neutralizing antibodies (bNAbs) summarized in this review.

Target Sites (See Figure 1)	bNAb	PDB Accession No. for Crystal Structure	Potency (Geometric Mean/Median IC_50_, μg/mL)	Breadth (% of *n* Pseudoviruses, IC_50_ < 50 μg/mL)	Research & Development Stage	References
CD4-binding	VRC01	3NGB	0.33/0.37	91% of 190	Phase I	[20,24]
site	3BNC117	4JPV	0.09/0.07	84% of 180	Phase II	[23]
V1/V2	PGDM1400	4RQQ	- */0.003	83% of 106	Preclinical	[27]
V3/Asn332	PGT121	4FQ1	0.05/0.022	64% of 177	Preclinical	[28,29]
glycan patch	10-1074	4FQ2	- **	57% of 119	Phase I	[28]
gp120/	PGT151	4NUG	- */0.008	66% of 117	Preclinical	[30,31]
gp41-	35O22	4TOY	0.056/0.033	62% of 181	Preclinical	[32]
interface	8ANC195	4P9M	- */0.85	57% of 118	Preclinical	[33,34]
MPER	10E8	4G6F	0.22/0.35	98% of 180	Preclinical	[35,36]

* The geometric mean IC_50_ is not available; ** The geometric mean and median IC_50_ are not available.
